# Antiproliferative and Morphological Effects of Fenretinide Lipid Nanosystems in Colon Adenocarcinoma Cells

**DOI:** 10.3390/pharmaceutics16111421

**Published:** 2024-11-06

**Authors:** Lorenzo Anconelli, Francesca Farioli, Pietro Lodeserto, Aikaterini Andreadi, Francesca Borsetti, Manuela Voltattorni, Lucrezia Galassi, Martina Rossi, Giovanna Farruggia, Paolo Blasi, Isabella Orienti

**Affiliations:** 1 Department of Pharmacy and Biotechnology, University of Bologna, Via San Donato 19/2, 40127 Bologna, Italy; lorenzo.anconelli3@unibo.it (L.A.); francesca.farioli@studio.unibo.it (F.F.); francesca.borsetti@unibo.it (F.B.); manuela.voltattorni@unibo.it (M.V.); lucrezia.galassi2@unibo.it (L.G.); martina.rossi12@unibo.it (M.R.); giovanna.farruggia@unibo.it (G.F.); 2 Section of Endocrinology and Metabolic Diseases, Department of Systems Medicine, University of Rome Tor Vergata, 00133 Rome, Italy; pietro.lodeserto@ptvonline.it (P.L.); andreadi@med.uniroma2.it (A.A.); 3 Center for Applied Biomedical Research (CRBA), University of Bologna, 40126 Bologna, Italy; 4 National Institute of Biostructures and Biosystems, Via dei Carpegna 19, 00165 Rome, Italy

**Keywords:** 4-hydroxyphenyl retinamide, all-trans retinoic acid, antitumor activity, quantitative phase imaging microscopy, mitochondrial membrane potential, reactive oxygen species, HT-29, colorectal cancer

## Abstract

**Objective:** Colon adenocarcinoma is characterized by the downregulation of the retinoic acid receptor, making natural retinoids such as all-trans retinoic acid, 9-cis retinoic acid and 13-cis retinoic acid effective in treatment and chemoprevention due to their ability to increase RARβ expression. However, major limitations to their use include tolerability and acquired resistance. In this study, we evaluated fenretinide, a semisynthetic derivative of all-trans retinoic acid, in an HT-29 cell line. Fenretinide was evaluated both as a free drug and encapsulated in self-assembling phosphatidylcholine nanosystems with the aim of increasing the aqueous solubility and cell availability of the drug. **Methods:** Fenretinide was encapsulated in lipid nanosystems obtained in water by the dispersion of an amphiphilic mixture of phospholipids, glyceryl tributyrate and polysorbate 80. The physico-chemical characterization of the nanosystems was carried out by dynamic light scattering and spectrophotometry. The biological activity was evaluated by quantitative phase imaging microscopy, MTT assay, flow cytometry and confocal laser-scanning fluorescence microscopy. **Results:** Fenretinide in phosphatidylcholine nanosystems was more active than free fenretinide in inhibiting HT-29 cells’ proliferation, as indicated by quantitative phase imaging data. Indeed, encapsulated fenretinide increased duplication time, decreased dry mass and decreased the rate of cell growth more efficiently than fenretinide. Moreover, encapsulated fenretinide effectively decreased the motility of the cells that survived the treatment. **Conclusions:** The results indicate that the proposed nanosystems can be considered a valuable alternative to natural retinoids in the chemoprevention and treatment of colorectal cancer. This is due to the favorable pharmacologic characteristics of fenretinide in colorectal cancer and the improved drug activity provided by nanoencapsulation.

## 1. Introduction

In colorectal cancers, the downregulation of retinoic acid receptors (RARs) (1.02 f mol/μg DNA in cancer cells with respect to 2.64 f mol/μg DNA in normal cells) [1] and the downregulation or loss of retinoid X receptor (RXR)-α have been largely demonstrated [2]. The retinoid signaling pathway is also deregulated. Two genes in particular, encoding for the enzymes retinol dehydrogenase 5 and retinol dehydrogenase-like, both involved in the biosynthesis of retinoic acid, were shown to be downregulated, resulting in colon tumor cells failing to convert retinol to its active metabolite, all-trans retinoic acid (ATRA) [3]. For these reason, natural retinoids, such as ATRA, 9-cis retinoic acid (9-cisRA), and 13-cis retinoic acid (13-cisRA), have been extensively studied and their chemo-preventive and therapeutic properties have been demonstrated against colorectal cancer (CRC) [3]. Their effect was attributed to increased RARβ expression in sensitive cells and the overexpression of RARβ in ATRA-resistant cells, restoring their sensitivity [3].

In several chemical-induced CRC mouse models, ATRA decreased malignant tumor formation and progression, inhibited the establishment and evolution of aberrant crypt foci and increased the expression of RARs. However, in preclinical studies, the administration of natural retinoids for a protracted period of time at doses consistent with tolerability in humans resulted in poor outcomes, mainly due to acquired resistance [4,5] and toxicity [3,6].

Fenretinide (Fen) is a semisynthetic derivative of ATRA endowed with antitumor activity in a wide range of tumor types [7] and a low toxicity profile. Fen antitumor activity is based on multiple mechanisms triggering apoptosis in tumor cells. Among them, reactive oxygen species (ROS) increase, imbalance in the ceramides/dehydroceramides ratio, mTOR inhibition and RARβ induction [8] have been recognized as the most important in Fen antitumor activity. In CRC cell lines, the Fen effect has mainly been attributed to interference with the ceramide pathway, which generates an increase in dihydrosphingolipids and sphinganine cell levels, leading to apoptosis, the inhibition of prostaglandins synthesis [9] and the induction of the transcription factor CCAAT/enhancer with the upregulation of the apoptotic death receptor [3,10]. Fen activity was demonstrated in colorectal cancer spheroids [11,12] and in mouse models of colon adenocarcinoma, where high antitumor efficacy was obtained without a significant effect on normal counterparts [12,13]. The efficacy and low toxicity profile of Fen have been proved by many preclinical and clinical studies on solid and hematological tumors. However, despite these positive characteristics, its clinical use has so far been prevented by its poor solubility in water, which makes its bioavailability insufficient to achieve a high therapeutic response rate. Many attempts have been undertaken to develop new Fen nanoformulations with the aim of increasing the drug’s aqueous solubility and consequently enhancing its availability both in body fluids and target cells [14,15,16,17,18,19]. In this study, we prepared a new Fen nanoformulation based on the self-assembling of phospholipids/tryglicerides/polysorbate/Fen that achieves the aqueous solubilization of Fen by its inclusion in lipid nanosystems. The nanoformulation, referred to as PCN-Fen, was evaluated in the colon adenocarcinoma cell line HT-29 in comparison with the free drug to assess if the advantages of increased drug solubilization due to nanoencapsulation were also matched with increased antiproliferative effects.

## 2. Materials and Methods

### 2.1. Chemicals

Fenretinide (N-4-hydroxyphenyl-retinamide) was purchased from Olon Spa (Milan, Italy); soy L-α-phosphatidylcholine, glyceryl tributyrate, polysorbate 80, KOH, dichlorofluorescein diacetate (DCHFDA) and Hoechst 33342 (HO) were from Sigma-Aldrich (Milan, Italy), while ethanol absolute anhydrous was purchased from Carlo Erba Reagents (Milan, Italy). Dulbecco’s Minimal Essential Medium High Glucose (DMEM), glutamine, trypsine/EDTA solutions and Fetal Bovine Serum (FBS) were from Gibco (Thermo Fisher, New York, NY, USA).

### 2.2. Preparation of PCN-Fen

Fen nanosystems were prepared according to a previously reported method [14,15] with some modifications. Briefly, soy phosphatidylcholine (4 mmoles), glyceryl tributyrate (2 mmoles), polysorbate 80 (0.12 mmoles) and KOH 10 N (400 µL, 4 mmoles) were mixed to homogeneity to obtain a semisolid phase. Fen (1.2 mmoles) was dissolved in ethanol (300 µL) and KOH 10 N (120 µL, 1.2 mmoles) and was subsequently added to the mixture. The resultant semisolid phase was dispersed in PBS pH 7.4 to 10 mg/mL, filtered through 0.2 µm filters and dialyzed for 72 h (dialysis membrane Mw cutoff 10 KD) against PBS pH 7.4. The dialyzed phase was lyophilized. The dry residue was reconstituted with water and filtered again through 0.2 µm filters to obtain 10 mg/mL PCN-Fen, representing the dispersion that was stored at −22 °C until use. Empty nanosystems (PCNs) were also prepared through the same procedure but without the addition of Fen.

### 2.3. Characterization of PCN-Fen

The loading and encapsulation efficiency of Fen in the nanosystems was evaluated as previously described [15,16,17]. Briefly, the reconstituted nanosystems’ dispersions (10 mg/mL) were diluted (1:3, *v*/*v*) with an ethanol:water (1:1, *v*/*v*) mixture and analyzed for drug content in comparison with the empty nanosystems. The content of Fen was obtained by UV spectroscopy (Shimadzu UV-1601, Kyoto, Japan) at 360 nm. Drug loading was calculated as the percentage weight of Fen in the nanosystems according to the following equation:

$$Drug\;Loading\;\left(\%\frac{w}{w}\right)=\left[\frac{weight\;of\;Fen\;in\;10\;mg\;PCN\_Fen}{10\;mg\;PCN\_Fen}\right]\times 100$$

The encapsulation efficiency was obtained as the percentage weight of Fen in the nanosystems with respect to that used in the preparative mixture according to the following equation:

$$Encapsulation\;Efficiency\;\left(\%\frac{w}{w}\right)=\left[\frac{weight\;of\;Fen\;in\;10\;mg\;PCN\_Fen}{weight\;of\;Fen\;added\;to\;10\;mg\;PCN\_Fen}\right]\times 100$$

The particle size and zeta potential of the reconstituted nanosystems were measured at 37 °C (Malvern Nano-ZS Spectrometer, Malvern, UK) at 10 mg/mL in pH 7.4 PBS. The effect of dilution on particle size was evaluated after dilution with PBS and PBS containing FBS (10% *v*/*v*) up to 0.01 mg/mL. A minimum of 12 measurements per sample were made. The results were the combination of 3–10 min runs for a total accumulation correlation function time of 30 min. The results were volume-weighted. The stability of the nanosystems to drug leakage was measured at 24 h by the evaluation of Fen released from PCN-Fen, as previously described [14,15,18,19], with some modifications. Briefly, the reconstituted nanosystems’ suspensions (10 mg/mL) were diluted to 1 mg/mL with pH 7.4 PBS containing FBS (10% *v*/*v*), and 1 mL of the diluted suspension was placed in a releasing chamber separated from a receiving compartment by a dialysis membrane (Mw cutoff 5 k Da, Fisher Scientific, Milan, Italy). The receiving compartment was filled with 10 mL pH 7.4 PBS containing FBS (10% *v*/*v*). The system was maintained at 37 °C, and sink conditions were monitored throughout the experiment. Leakage from the nanosystems was evaluated at 24 h by the spectrophotometric determination of Fen in the receiving phase at its maximum absorption wavelength (360 nm) by the following equation:

$$Drug\;Leakage\;at\;24\;h\;\left(\%\right)=\left[\frac{amount\;of\;Fen\;in\;the\;receiving\;phase\;at\;24\;h}{amount\;of\;Fen\;loaded\;in\;1\;mg\;PCN\_Fen}\right]\times 100$$

### 2.4. Cell Lines

HT-29 cells were provided by ATCC (Manassas, VA, USA). For the present study, HT-29 cells were grown in DMEM supplied with 10% FBS, penicillin (100 UI/mL) and streptomycin (0.1 mg/mL) at 37 °C in a 5% CO_2_-humidified atmosphere. They were maintained in 25 cm^2^ culture flasks (Corning, Tewksbury, MA, USA) and harvested using 0.25% Trypsin in 0.2 g/L EDTA solution in isotonic NaCl.

### 2.5. MTT Assay

To evaluate the effect of Fen or PCN-Fen on cell viability, the cells were treated and MTT assays were performed. Treatments were performed at increasing Fen concentrations and at PCN-Fen concentrations corresponding to the same concentrations as the free drug. The empty nanosystems were also tested at the same concentrations as the loaded ones. The tetrazolium salt 3-(4,5-dimethylthiazol-2-yl)-2,5-diphenyl tetrazolium bromide (MTT) assay was used to estimate cell proliferation and viability. Briefly, this assay is based on the reduction of MTT to insoluble formazan salt via cellular dehydrogenase. For this reason, the amount of formazan produced is considered a good indicator of the number of viable cells in the sample [20,21]. To perform the MTT assay, the cells were seeded at 1 × 10^4^ cell/cm^2^ in 96-well plates, and, after 24 h, they were treated with Fen, PCN-Fen or the empty nanosystems for 24, 48 or 72 h at concentrations of Fen ranging between 0.03 and 50 µM. Empty nanosystems were added in the same amount as the loaded ones. Afterwards, 10 µL of a 5 mg/mL MTT solution was added to each well to a final concentration of 0.5 mg/mL. After 4 h at 37 °C in the dark, the medium was removed and 100 µL of isopropanol was added to each well to dissolve the cells and to solubilize the formazan produced. The absorbance of each well was read on a TECAN plate reader (Männedorf, Switzerland) at 570 nm.

### 2.6. Quantitative Phase Imaging Microscopy

**Figure 1:**
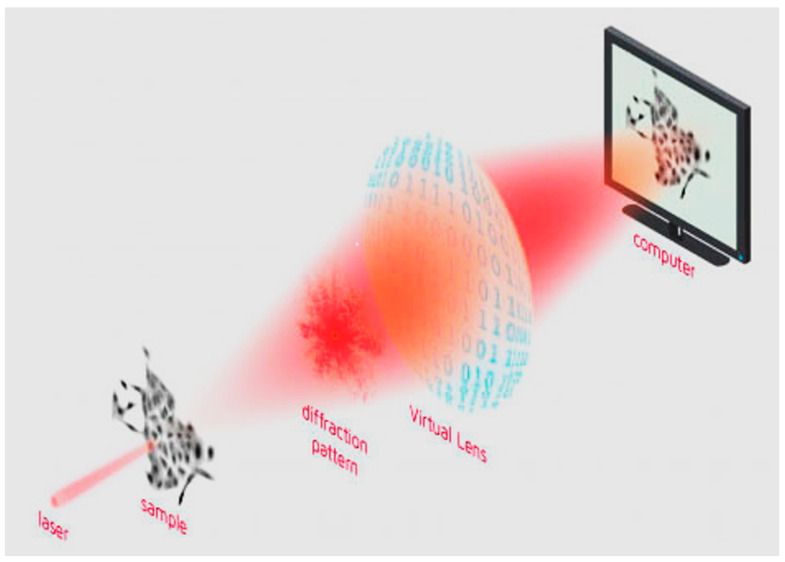
Schematic representation of image formation with the technology known as “ptychography”, a form of quantitative phase imaging (QPI). Reproduced with permission from https://www.phasefocus.com/about/technology, accessed on 30 October 2024.

For quantitative phase imaging (QPI) analysis, cells were seeded in a 96-well plate (Corning, Tewksbury, MA, USA) at 1 × 10^4^ cell/cm^2^. After 24 h, the cells were treated at the same concentrations as those used for the MTT assays. QPI was performed by a Livecyte^®^ Live Imaging System (Phasefocus, Sheffield, UK). Images were acquired every 60 min for 3 days using a 10× objective (0.25 NA) at 37 °C and 5% CO_2_. QPI data were analyzed using Cell Analysis Toolbox software version 3.8.1 (Phasefocus, Sheffield, UK). We evaluated cell confluence, thickness, cell displacement and instantaneous velocity to assess the treatments’ effects on cell proliferation, morphology and spreading ability. These effects were studied through image acquisition and analysis. These studies were performed using the Livecyte^®^ Live Imaging System and related software, capable of analyzing the images acquired both at the population and at the single-cell level. The technique used by the instrument is ptychography (ptychographic QPI—quantitative phase imaging), which is based on light diffraction. In particular, the sample to be analyzed is illuminated in a series of spots, and the light diffraction patterns are collected and analyzed. The collection is operated by a virtual lens, consisting of a sCMOS camera capable of transmitting the detected light diffraction pattern to a computer. A schematic representation of the image formation by ptychography is shown in Figure 1 [22].

### 2.7. Confocal Laser-Scanning Fluorescence Microscopy

Confocal laser-scanning microscopy (CLSM) is a valuable tool for imaging living and fixed specimens containing fluorophores. High-resolution images are generated by the superposition of photons emitted from the fluorophore that reach the detector during one exposure period. To image by CLSM the effects of the treatments on cell membranes, the cells were seeded in 4-well chamber slides (Lab-Tek Chambered Coverglass, Thermo Fisher, New York, NY, USA) at 1 × 10^4^ cell/cm^2^. After 24 h, they were treated with Fen or PCN-Fen. HO was added at 1 µg/mL and the cells were incubated at 37 °C in the dark. After 15 min, DiO was added at 10 µg/mL and, 15 min later, the cells were washed with PBS and fixed with paraformaldehyde (PFA) 2%. After 30 min, the slides were washed 3 times with PBS/glycine 0.1 M. HO and DiO fluorescences were excited at 405 nm and 488 nm, and emissions were recorded at 450/35 nm and 515/30 nm, respectively.

To evaluate the cell uptake of nanosystems, the cells were treated with PCN-Fen and Fen at *IC_50_* for 24 h. Afterwards, they were incubated for 1 h with 1 μg/mL HO to stain the cell nuclei. Subsequently, the cells were washed with PBS and incubated for 30 min with Nile Red Staining Solution 5 μg/mL. Finally, the cells were washed with PBS three times and fresh medium was added. The images were obtained as previously described. The HO and Nile Red fluorescences were excited at 405 nm and 543 nm, and emissions were recorded at 450/35 nm and 650 nm, respectively.

All the images were analyzed by Image J Software (version 1.53a, U. S. National Institutes of Health, Bethesda, MD, USA).

### 2.8. Flow Cytometry

Flow cytometry allows the analysis and distinction of different cell populations contained in a sample. Through this technique, the effects of Fen and PCN-Fen on cell cycle, mitochondrial potential and ROS production were evaluated. To prepare for the analysis, 3 Petri dishes containing approximately 1.7 × 10^6^ cells each were initially set up. The following day, when the cells were adherent, the medium contained in the plates was eliminated and replaced with 10 mL of pure medium or medium containing 10.75 μM of Fen or 11 μM of PCN-Fen. After 48 h, the cells were detached, centrifuged and resuspended in Hanks Balanced Salt Solution (HBSS) at a concentration of 5 × 10^5^ cells/mL.

For the analysis, a Sorter S3 flow cytometer (BioRad, Hercules, CA, USA) equipped with an Argon laser and ProSort software version 1.6 was used. The instrument was set to excite cells at 488nm and analyze the fluorescence emission in green (525 nm) and red (600 nm).

Negative control samples, containing the cells in HBSS without probes, were prepared to highlight the signals due to autofluorescence.

#### 2.8.1. Cell-Cycle Analysis

For cell-cycle analysis, cells were centrifuged and resuspended in an aqueous solution containing the following ingredients: propidium iodide (PI) 50 μg/mL, capable of stoichiometrically binding DNA and, therefore, quantifying it, allowing us to understand which phase of the cell cycle the cells are in; RNase 10 μg/mL, which digests the RNA so that the analysis is not distorted by the binding of PI to RNA; NP40 0.1%, detergent that allows the solubilization of the membranes; and citrate 0.1%, a hypotonic solution that promotes the lysis of cell membranes but stabilizes isolated nuclei. PI emits fluorescence in red at around 600 nm. The fluorescence was analyzed on a linear scale and the distribution of cells in the different phases of the cycle was analyzed with FCSalyzer software, version number 0.9.22-alpha.

#### 2.8.2. Mitochondrial Potential Analysis

For the analysis of mitochondrial potential, a 5,5′,6,6′-tetrachloro-1,1′,3,3′-tetraethylbenzimidazolyl-carbocyanine iodide (JC1) probe was used. This is a cationic probe capable of accumulating in compartments rich in negative charges, such as the matrixes of active mitochondria. At low concentrations, the probe is solubilized in its monomeric form, characterized by a green fluorescence. At high concentrations, such as those generated by accumulation in healthy active mitochondria, the probe molecules aggregate, shifting the fluorescence emission from green to red. Conversely, in suffering mitochondria, characterized by low membrane potential, the concentration of the probe decreases, shifting the fluorescence emission from red to green. This allows us to evaluate the health status of the mitochondria and their potential. For this assay, 5.0 × 10^5^ cells/mL were incubated with 2 μM JC1 for 15 min at 37 °C in the dark, and they were subsequently analyzed through the QuadStat application by acquiring the two fluorescences on a logarithmic scale.

#### 2.8.3. Evaluation of Reactive Oxygen Species Production

The analysis of ROS production was carried out using the non-fluorescent probe DHCFDA, which is converted into fluorescent species by the concomitant action of ATP-dependent cellular esterase and oxidation by ROS. In this way, DCF, which emits green fluorescence at 425 nm, is formed. The cells were washed and incubated with 2 μM DHCFDA for 30 min at 37 °C, in the dark and in the presence of 5 μg/mL PI, which allows the dead cells to be highlighted. The samples were then centrifuged at 200× *g* for 10 min and resuspended in HBSS buffer in order to eliminate excess probe. The two fluorescences were acquired on a logarithmic scale and the fluorescence distribution of live cells was analyzed.

### 2.9. Statistical Analysis

All experiments were repeated at least three times on three independent samples. One-way analysis of variance (ANOVA) followed by Dunnett’s multiple comparison test was used for repeated measurement values. Differences of *p* < 0.05 were considered significant.

Statistical analysis was carried out using GraphPad Prism Software (version 6.0c, GraphPad Software, San Diego, CA, USA). One-way ANOVA with Tukey’s multiple comparison test was used. Group differences were considered statistically significant when *p* < 0.05.

## 3. Results

### 3.1. PCN-Fen Characteristics

PCN-Fen was obtained by the dispersion in PBS of the semisolid mixtures prepared by mixing phosphatidylcholine, glyceryl tributyrate, polysorbate 80 and Fen. The spontaneous self-assembling of the mixture components in the aqueous phase provided glyceryl tributyrate nanosystems stabilized by phosphatidylcholine and polysorbate 80, trapping Fen in their lipophilic core. The physicochemical parameters of PCN-Fen revealed the suitability of these systems for the effective incorporation of Fen, with drug loading at about 11% (*w*/*w*) and an encapsulation efficiency higher than 95% (*w*/*w*) (Table 1).

PCN-Fen nanosystems show a slightly negative charge, as indicated by their zeta potential. Drug leakage was about 6% at 24 h, indicating the good drug retention ability of PCN-Fen in an aqueous environment at physiological pH. This is an essential feature in antitumor therapy to avoid drug release in circulation and maximize on-target release at the tumor site.

**Figure 2:**
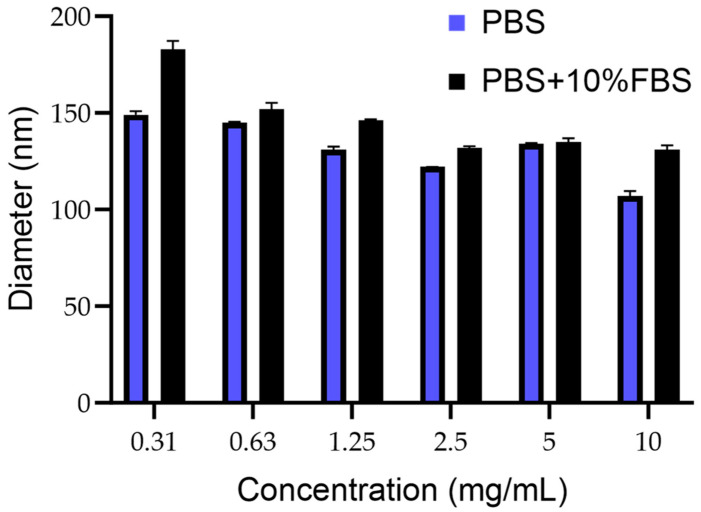
Mean diameter of PCN-Fen (nm) at different concentrations in PBS pH 7.4 and PBS containing FBS 10% (*v*:*v*).

The mean size of PCN-Fen was lower than 200 nm in the whole concentration range examined (10 mg/mL–0.31 mg/mL), which was representative of possible formulative concentrations and their progressive dilution in blood after administration (Figure 2).

The size being under 200 nm makes PCN-Fen suitable for tumor target therapy. Indeed, the extravasation of nanosystems through the discontinuities of tumor capillaries, which generates selective drug accumulation at the pathological site, has been demonstrated to be highly improved by decreasing nanoparticle dimensions to below 200 nm [23,24].

The size constancy after dilution (Figure 2) is another favorable feature, indicating the structural stability of PCN-Fen against swelling, disassembly, and/or reorganization. In flexible/deformable structures, such as liposomes and nanoemulsions, dilution with blood after injection can trigger these very dangerous phenomena [25,26]. Indeed, the Food and Drug Administration requires, for liposomes, product stability validation that includes dilution studies to demonstrate the system’s stability (size/morphology and structure) under the real conditions of use.

Furthermore, the limited size increase in PCN-Fen in the presence of serum (Figure 2) indicates a reduced protein corona effect on the PCN-Fen surface, as expected by its negative zeta potential and highly hydrophilic surface, limiting the absorption of serum proteins. On the other hand, the protein corona is known to have detrimental effects on nanosystems. Protein adsorption increases the nanosystem size and masks the surface features compromising their ability to extravasate to the tumor site and target specific receptors [27].

### 3.2. Effect of Fen and PCN-Fen on Cell Viability

**Figure 3:**
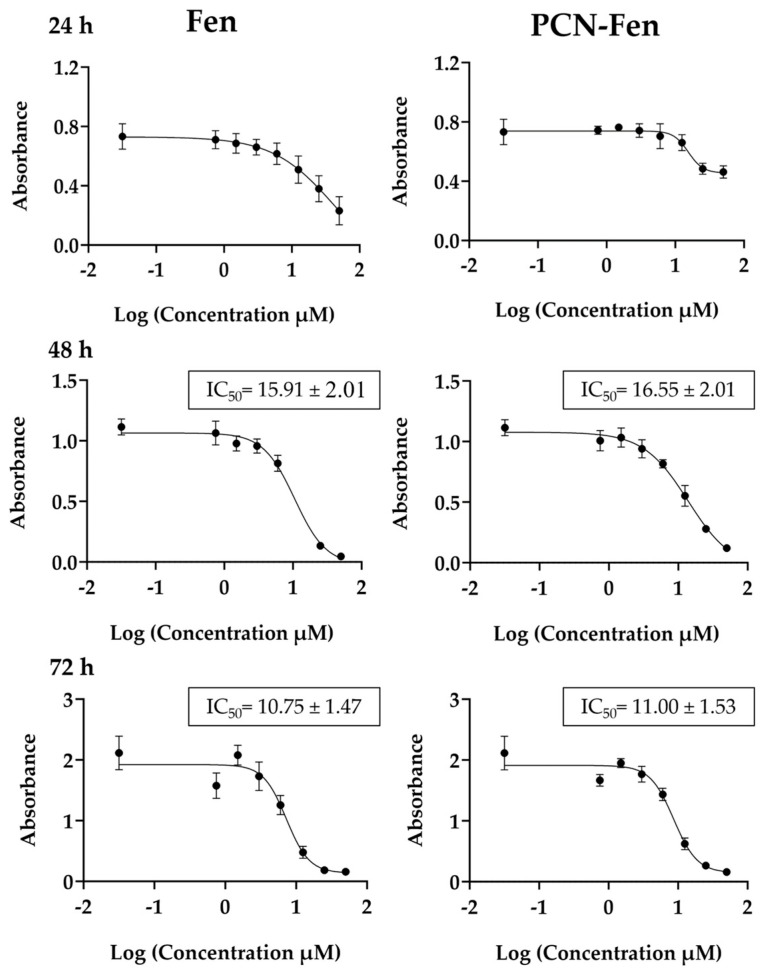
Viability of cells treated with Fen and PCN-Fen at different drug concentrations at 24 h, 48 h and 72 h, determined by MTT assay.

MTT assays were performed on Fen and PCN-Fen-treated cells at Fen concentrations in the range of 0.03–50 µM, corresponding to nanosystem concentrations in the range of 0.11–181.3 µg/mL for different time periods (Figure 3).

Cytotoxicity was expressed as the *IC_50_* value, which is the concentration that reduces cell viability by 50%. The results, reported in Figure 3, were determined at 48 and 72 h. At 24 h, treatment with Fen and PCN-Fen provided a limited cytotoxic effect, as revealed by the reduction in MTT, which, even at the highest concentrations, was only 25% compared to the control. Therefore, at 24 h, *IC_50_* could not be determined because sufficient mortality was not achieved to calculate it correctly. The *IC_50_* values did not differ significantly between PCN-Fen and Fen, indicating that nanoencapsulation did not modify drug efficacy. This finding was confirmed by the lack of cytotoxicity of the empty nanosystems at the concentrations corresponding to the *IC_50_* values for the Fen-loaded ones. Throughout this work, we have used the *IC_50_* values at 72 h and, as a comparison, we have also used the half and double values, indicated, respectively, as *0.5-IC_50_* and *2-IC_50_*.

### 3.3. Quantitative Phase Imaging

The evaluation of the cellular response to the treatments by MTT is mainly based on mitochondrial activity and cell metabolic status. The results are averaged over the entire cell population and may therefore not discriminate among different metabolic effects [28]. Furthermore, the MTT tests are performed at discrete time intervals with loss of the temporal dynamics of phenomena such as cell death and morphological alterations, which can lead to progressive cellular deterioration and unexpected outcomes.

Time-lapse microscopy, which uses microscopes equipped with incubators, can provide more accurate information. Time-lapse microscopy techniques, such as QPI, evaluate cell morphology over time and measure parameters such as cell confluence, doubling time, shape, sphericity, cell displacement, and instantaneous velocity in response to treatment. In this study, Livecyte^®^, a live imaging system was used. This instrument uses light diffraction to reconstruct cellular images without requiring fluorescent labels, as in fluorescence microscopy, or lenses that can cause aberrations, as in phase contrast optics. Furthermore, it is possible to obtain an estimation of the dry mass of the population or the individual cells. We treated the cells with Fen and PCN-Fen for 72 h at the respective *IC_50_, 0.5-IC_50_* and *2-IC_50_* values. The data obtained were analyzed to extrapolate the parameters described in detail in the following sections.

#### 3.3.1. Cell Proliferation

**Figure 4:**
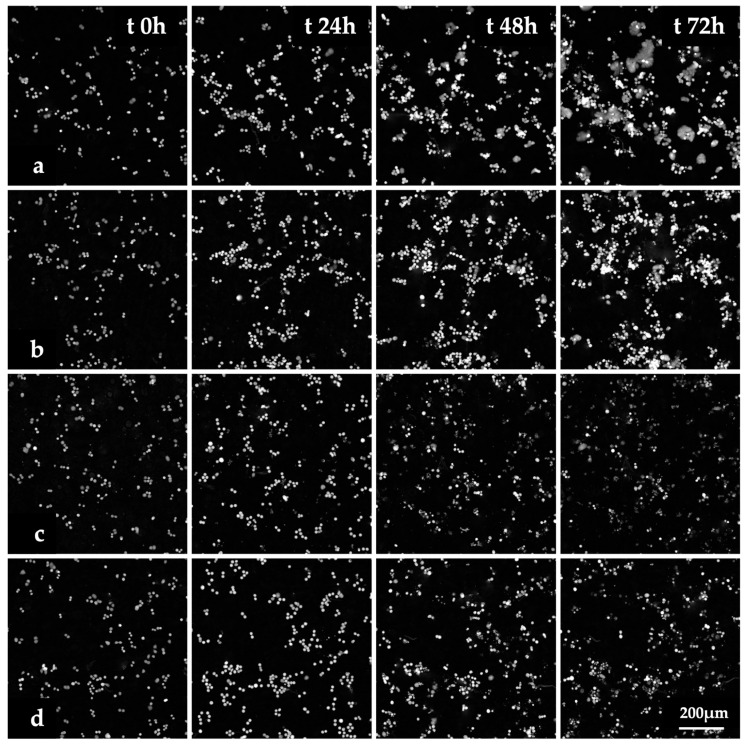
Images of HT-29 cells obtained by QPI at various time points. Controls (**a**) and cells treated with PCN (**b**), Fen (**c**) and PCN-Fen (**d**) at *IC_50_*.

The images of HT-29 cells treated with Fen or PCN-Fen at *IC_50_* at different time points (Figure 4) and the videos of cell proliferation over time up to 72 h (Videos S1–S4) indicated that treatment with Fen and PCN-Fen significantly decreased cell viability with respect to the controls, while PCN did not affect cell viability. No appreciable differences were shown between Fen and PCN-Fen.

**Figure 5:**
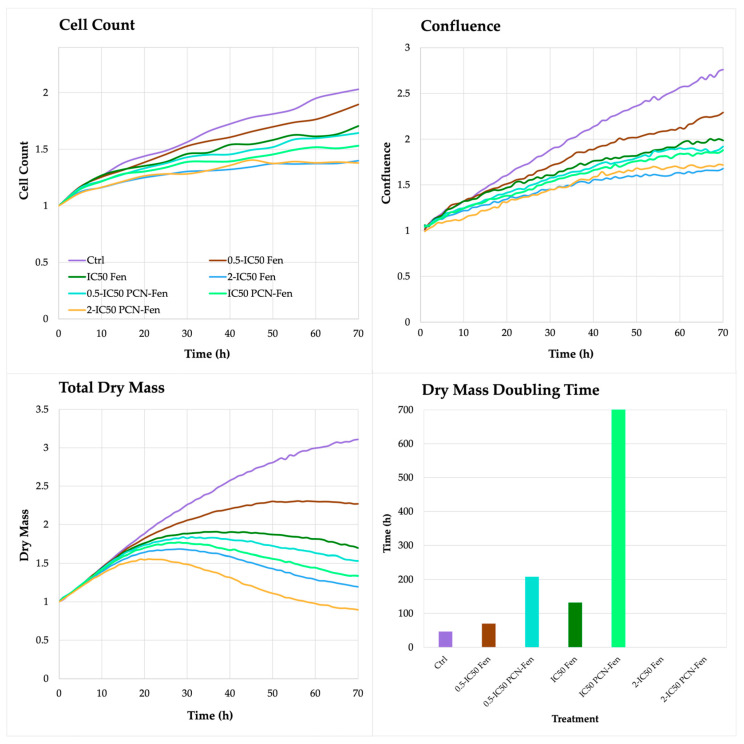
Cell count, confluence, total dry mass and dry mass doubling time of treated cells, determined by QPI.

On the contrary, the QPI parameters related to cell proliferation, such as cell count, confluence, total dry mass and dry mass doubling time, revealed significant differences between treatments. In untreated HT-29, the cell count, confluence and total dry mass increased up to 72 h, with a rate that was almost constant over time. Treatment with Fen and PCN-Fen significantly decreased cell count, confluence and dry mass with respect to the controls (Figure 5).

The differences between the treatments and the control progressively widened over time. In particular, cell number and confluence were comparable in the treated cells and controls in the first 24 h, while significant differences were obtained at 72 h. The dry mass progressively increased in untreated cells up to 72 h. In treated cells, on the contrary, the dry mass grew until 24 or 48 h, and subsequently decreased up to 72 h (Figure 5).

This behavior confirmed the limited efficacy of treatments in the first 24 h, as observed by the MTT assay. Fen and PCN-Fen at *0.5-IC_50_* increased the dry mass at a comparable rate with the controls until 24 h. After that, a slowdown in the dry mass increase was observed in Fen and a plateau was observed in PCN-Fen up to 48 h, followed by a decrease in dry mass up to 72 h (Figure 5).

The treatments at *IC_50_* and *2-IC_50_* provided the strongest effect, with a significant decrease in dry mass just after 24 h, suggesting that the cells lost a good part of their internal content. This was confirmed by the duplication time of the dry mass calculated by the software for each treatment at each concentration. The doubling time of the dry mass of cells treated with Fen at *0.5-IC_50_* was similar to the control, while at *IC_50_* it increased, indicating an improved effect of treatment. At *IC_50_*, the doubling time of the dry mass was not calculable anymore because a marked decrease in cell population, compared to the inoculum, made impossible the evaluation of this parameter. A similar trend was observed with PCN-Fen but, in this case, the doubling times of the dry mass were considerably longer than those obtained with Fen, indicating a stronger cytotoxic activity of PCN-Fen than Fen. Indeed, treatment with PCN-Fen at *0.5-IC_50_* raised the dry mass doubling time to about 200 h, corresponding to about 4 times the doubling time of the controls (50 h); treatment at *IC_50_* increased the dry mass doubling time to about 700 h; and, again, at *2-IC_50_*, the doubling time of the dry mass was not calculable because of the marked decrease in cell population.

#### 3.3.2. Cell Morphology

Cell thickness, size and sphericity are parameters related to cell morphology provided by QPI. They give information about the state of health, sufferance, or epithelial–mesenchymal transition (EMT) of cells after treatment. Cell sufferance can either provide phenotypes characterized by increased thickness, sphericity and size, with cells that tend to detach from the substrate, or phenotypes with decreased thickness, sphericity and constant area, with cells that remain attached to the substrate [29]. The EMT of HT-29 cells has been described in the literature as characterized by increased cell thickness, sphericity and cell size [30].

**Figure 6:**
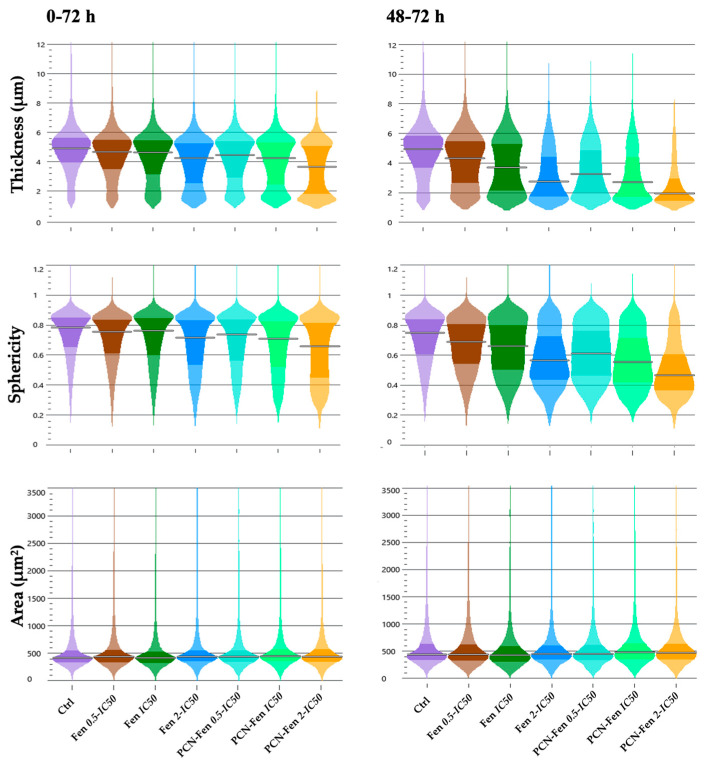
Thickness, sphericity and area of treated cells at different time intervals: 0–72 h and 48–72 h (determined by QPI). Statistical analysis revealed significant differences between treatments at all the concentrations analyzed, except for the area evaluation.

Treatments with Fen and PCN-Fen decreased thickness and sphericity without significant variations in cell area, which correlates with the observed decrease in dry mass and indicates sufferance. PCN-Fen’s effects were greater than Fen’s, with differences becoming wider in the 48–72 h interval (Figure 6).

#### 3.3.3. Cell Motility

**Figure 7:**
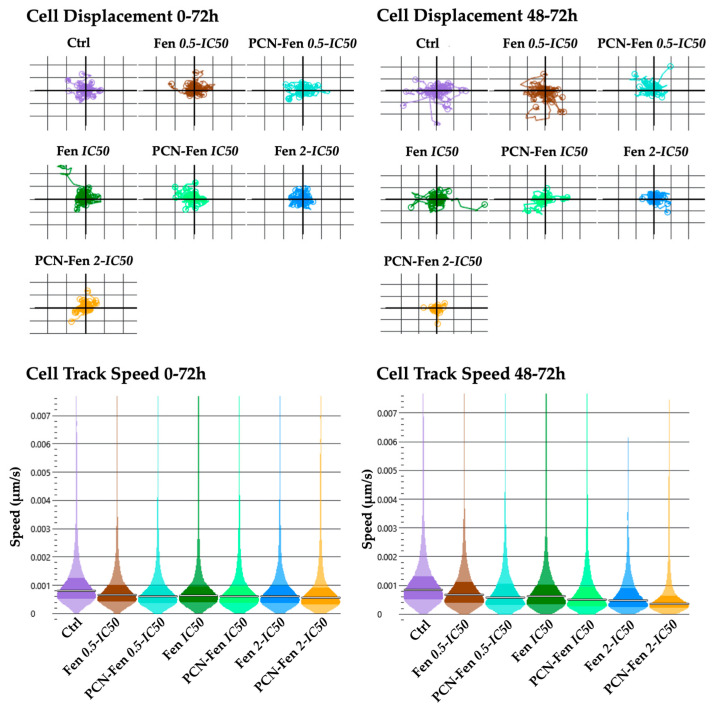
Cell displacement and track speed at different time intervals: 0–72 h and 48–72 h (determined by QPI). Statistical analysis revealed significant differences between treatments at all the concentrations analyzed.

Cell displacement and track speed were the parameters related to cell motility provided by QPI. The measures were obtained in the whole period of treatment (0–72 h) and in the 48–72 h interval, which corresponded to the maximum activity of the treatments, as revealed by the other QPI parameters related to cell proliferation. Cell displacement and track speed were decreased by the treatments, with a higher effect in the 48–72 h period. PCN-Fen was more active than Fen at comparable concentrations. An almost complete suppression of cell displacement and speed was observed with PCN-Fen at *2-IC_50_* in the 48–72 h period, indicating the superiority of the nanoformulation with respect to the free drug in restraining tumor cell motility, and therefore the invasiveness correlated to cancer pathology (Figure 7).

### 3.4. Confocal Fluorescence Microscopy

**Figure 8:**
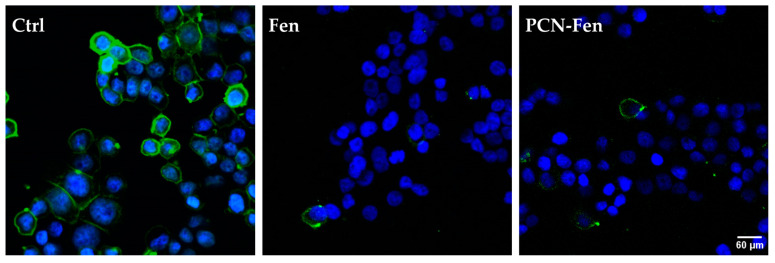
Confocal microscopy images of cells stained with the fluorescent dye DiO specific for the cell membranes, showing no fluorescence in treated cells. The nuclei are stained with HO.

HT-29 cells, grown on slides, were treated with Fen or PCN-Fen at high concentrations (37.5 μM Fen) for 24 h. Subsequently, the cells were labeled with HO to stain the nuclei and with DiO to stain the membranes and analyzed by confocal microscopy. The images revealed stained membranes in the untreated cells (green), while no stain was observed in the membranes of the treated cells (Figure 8). This suggested that a modification in the plasma membrane was induced by the treatment, hindering the penetration of the lipid DiO dye into the phospholipid bilayer. This hypothesis correlates well with the demonstrated ability of Fen to inhibit dihydroceramide desaturase [31,32,33], thus increasing the production of dihydroceramide compared to ceramides and consequently increasing the rigidity of the cell membrane due to the higher presence of saturated sphingolipids. 

**Figure 9:**
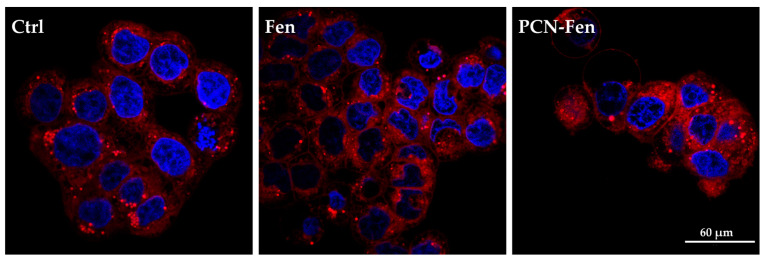
Confocal images of cells stained with Nile Red. The nuclei are stained with HO.

HT-29 cells are rich in lipid droplets, which can be stained by Nile Red: images of the stained cells are reported in Figure 9.

Treatment with PCN-Fen increased the red fluorescence of the cells to a higher extent than with Fen. This indicates the enhanced cell penetration of the dye, which could be correlated to the presence of the lipophilic PCN-Fen in the cells increasing the lipophilic character of the cytoplasm, and therefore its affinity towards lipophilic substances.

### 3.5. Flow Cytometry Data

**Figure 10:**
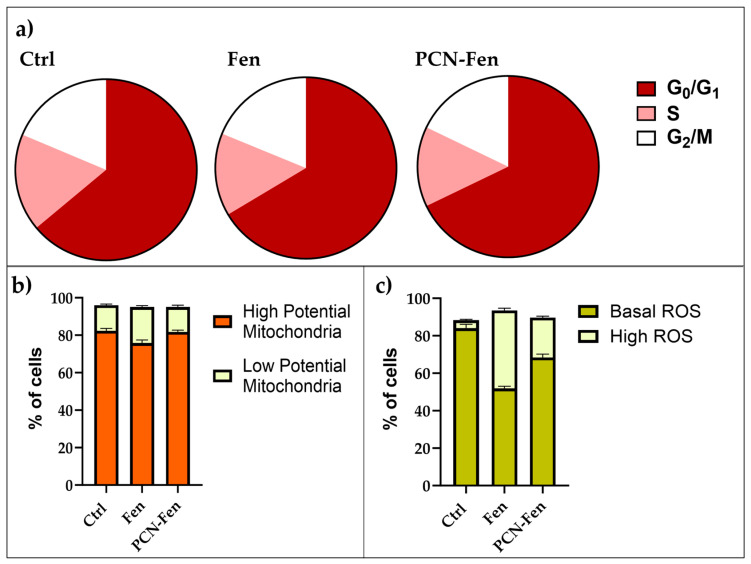
Comparison of flow cytometric evaluation of cell cycles (**a**), mitochondrial status (**b**) and ROS production (**c**).

Flow cytometry was used to evaluate the effects of Fen and PCN-Fen treatment at *IC_50_* for 48 h on cell-cycle progression, mitochondrial activity, ROS production and membrane labelling (Figure 10). The cytograms of the cells treated with PCN-Fen and PCN are reported in the Supplementary Materials (Figures S1–S3).

#### 3.5.1. Cell-Cycle Analysis

Treatment with Fen and PCN-Fen did not significantly affect cell-cycle progression, with the percentages of treated cells in the different phases of the cell cycle being similar to the controls (Figure 10a)

#### 3.5.2. Mitochondrial Potential

Mitochondrial potential was not significantly modified by the treatments. The percentages of cells with high or low mitochondrial potential were similar to the controls after treatment with either Fen or PCN-Fen (Figure 10b).

#### 3.5.3. ROS Production

ROS increase was observed in the treatments with Fen and PCN-Fen. The percentages of cells characterized by basal and high ROS levels, obtained from the cytograms, are reported in Figure 10c. Treatment with Fen and PCN-Fen significantly increased the high ROS cell population in accordance with the demonstrated ability of Fen to interfere with the protein kinase Cδ (PKCδ) in the mitochondrial signalosome, leading to the overactivation of oxidative phosphorylation [34]. The lower ROS increase provided by PCN-Fen, with respect to Fen, can be attributed to the delayed release of Fen from the nanosystems. This is a positive feature of nanoformulations containing antitumor drugs, as it avoids a sudden drug release during circulation and allows for a more efficient on-target release at the tumor site to be achieved [35].

## 4. Discussion

The antitumor activity of Fen has been largely proven in a wide range of hematological malignances and solid tumor types, including colorectal cancers [3,4,5,6,7,8,9,10,11,12,13]. However, despite its good activity and low toxicity profile, demonstrated also in clinical trials, Fen has not yet entered clinical use because its very low bioavailability prevents a high response rate, and the conventional formulations currently used for this drug have not solved this problem. Nanotechnology-based formulations have shown the possibility of increasing Fen bioavailability in animal models of colon cancer and lung cancer xenografts compared with conventional formulations [16]. The mechanism of this increased bioavailability involves Fen entrapment in supramolecular aggregates that enhance drug solubilization in aqueous body fluids and intracellular drug penetration [12,14]. In this study, we prepared PCN-Fen, a new nanoformulation based on a mixture of phospholipids/glyceryl tributyrate/polysorbate 80/Fen that is able to self-assemble in water with the formation of nanosystems that entrap Fen in their lipophilic core. Compared to other nanosystems reported in the literature, PCN-FEN showed higher drug loading [14,16,17,18,19], higher encapsulation efficiency [15,16] and smaller size [14,16,18,19]. PCN-Fen was evaluated in the colon adenocarcinoma cell line HT-29 in comparison with free Fen with the aim of predicting a possible transition of this new nanoformulation to preclinical studies. We found that Fen and PCN-Fen were cytotoxic towards HT-29 with comparable activities, with *IC_50_* values being about 16 μM and 11 μM for both formulations at 48 and 72 h, respectively. These values, obtained by MTT assay, indicated that both treatments induced a similar decrease in the metabolic activity of the cell population at the time points analyzed. However, MTT did not provide further information on the effects on the single cells at morphological and functional levels. These are important data to know as they allow the evaluation of the overall effectiveness of a treatment and its ability to prevent the recurrence of disease.

QPI is a new technique that provides detailed information on the antiproliferative effect of a treatment over time. Moreover, QPI can analyze multiple morphological displacement and growth parameters, whose combination allows the prediction of whether the cells that survived treatment have the potential to resume proliferation.

In the present study, the QPI parameters related to cell viability, such as cell count, cell confluence, dry mass and dry mass doubling time, all indicated a higher antiproliferative effect of PCN-Fen than Fen. The QPI parameters related to cell morphology further confirmed these findings by revealing decreased sphericity and thickness and a constant area of the cells treated with PCN-Fen. These morphological variations, together with the decreased dry mass, indicated sufferance in the cells that survived the cytotoxic effects of the treatment. The degree of sufferance in survived cells is an important indicator of the efficacy of antitumor therapy, since these cells can either die or resume tumor growth over time, thus generating a relapse of disease. PCN-Fen is expected to provide substantial tumor inhibition in vivo, as suggested by the decreased dry mass in the surviving cells, which can trigger senescence, followed by quiescence or spontaneous cell death [36].

Another effect of treatment was the inhibition of cell motility. The QPI parameters related to cell motility, such as displacement and track speed, indicated that PCN-Fen was more effective than Fen in decreasing both cell displacement and track speed, thus raising the possibility that PCN-Fen may hinder the migration of tumor cells that survive treatment. This is another important aspect in antitumor therapy, as the cells that survive chemotherapeutic treatments often acquire metastatic potential and resume tumor growth at distant sites that are often inaccessible to systemic therapy, thus causing failure of treatment. Finally, the superior activity of PCN-Fen over Fen in the HT-29 cells, related to the ability of the nanosystems to transport high drug cargo into the cells, was more evident in the 48–72 h time interval after treatment. This is due to the delayed release of the drug from the nanoformulation, which delays the onset of activity related to the amount of drug transported into the cells by the nanosystems. On the other hand, delayed drug release is an advantage in anticancer therapy since it avoids sudden drug release into the blood during circulation and provides efficient targeted release at the tumor site after extravasation.

## 5. Conclusions

Fen can be considered a suitable drug in colon adenocarcinoma since this disease is characterized by the downregulation of the retinoic acid receptor, and Fen has been proved to increase RARβ expression in a wide range of cancer cells, including colon adenocarcinoma. The encapsulation of Fen in lipid nanosystems provided a new nanoformulation, PCN-Fen, endowed with physico-chemical characteristics suitable for drug transport to the tumor site and on-target release. The in vitro studies in HT-29 cells demonstrated a higher antiproliferative activity of PCN-Fen than the free drug. PCN-Fen also affected the cells that survived treatment by decreasing their motility and their dry mass, thus suggesting an effect that can last beyond treatment, avoiding the possibility of metastatic spreading and the relapse of disease. Based on these results, preclinical studies are warranted.

## Figures and Tables

**Table 1:** Drug loading, encapsulation efficiency, zeta potential and drug leakage of PCN-Fen in pH 7.4 PBS.

Formulation	Drug Loading (% *w*/*w*)	Encapsulation Efficiency (% *w*/*w*)	Zeta Potential (mV)	Drug Leakage at 24 h (%)
PCN-Fen	10.8 ± 0.9	95.2 ± 3.2	−17.1 ± 0.6	5.9 ± 1.8

## Data Availability

All data are available on request to the corresponding authors.

## Supplementary Materials

The following supporting information can be downloaded at: https://www.mdpi.com/article/10.3390/pharmaceutics16111421/s1, Figure S1: Cell-cycle analysis flow cytometric plots; Figure S2: Mitochondrial potential evaluation flow cytometric plots; Figure S3: ROS production analysis flow cytometric plots; Video S1: Control cells’ proliferation; Video S2: PCN-treated cells’ proliferation; Video S3: Fen-treated cells’ proliferation; Video S4: PCN-Fen-treated cells’ proliferation.
