# Supplementary material for: Antiproliferative and Morphological Effects of Fenretinide Lipid Nanosystems in Colon Adenocarcinoma Cells
Source: Pharmaceutics. 2024 Nov 6;16(11):1421. doi: 10.3390/pharmaceutics16111421 (PMC11597870; doi:10.3390/pharmaceutics16111421)
Supplement: Supplementary file 1 [file pharmaceutics-16-01421-s001.zip › pharmaceutics-3256385-supplementary.pptx]

## Slide 1
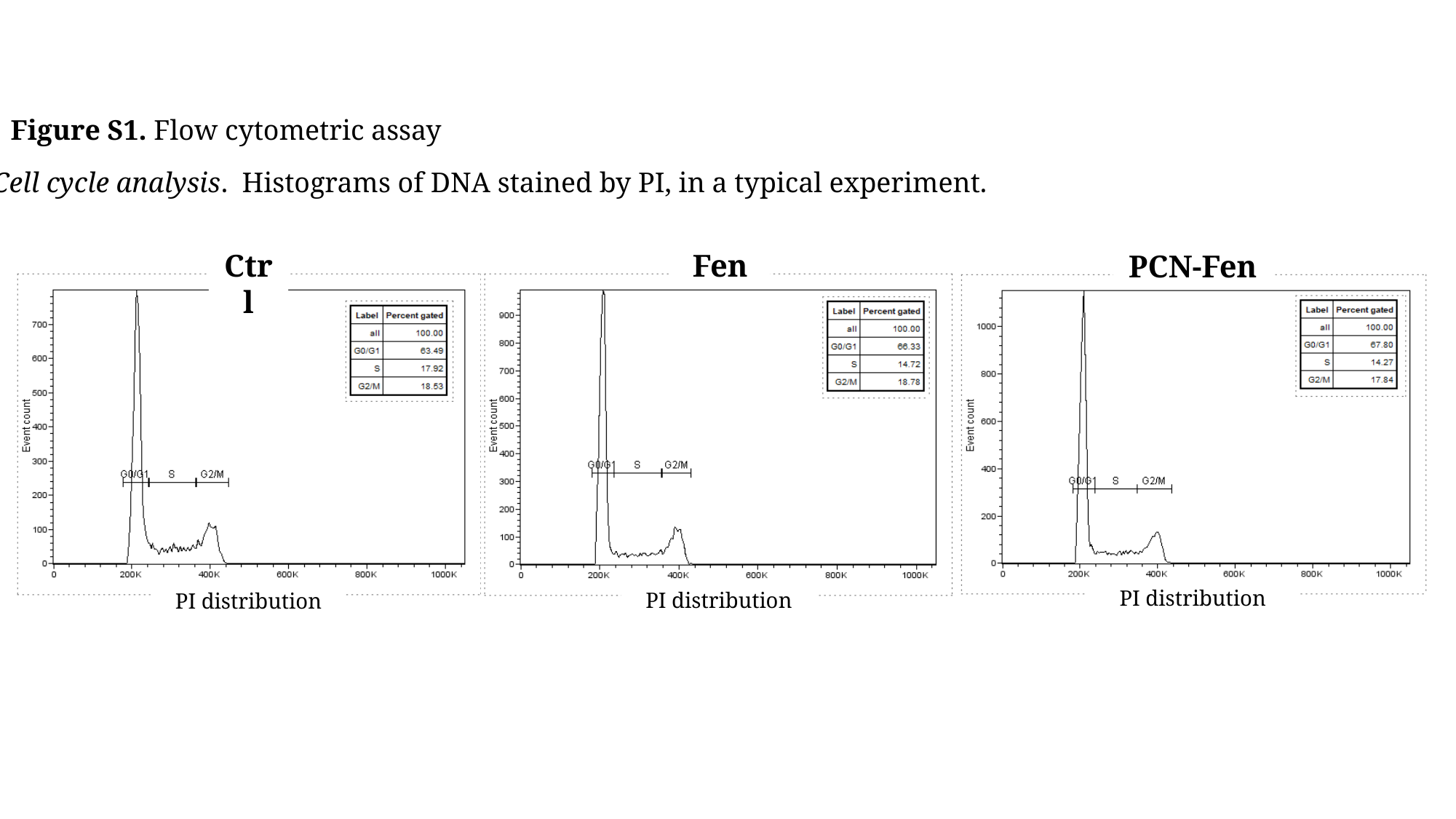

Figure S1. Flow cytometric assay
Cell cycle analysis. Histograms of DNA stained by PI, in a typical experiment.
Ctrl
Fen
PCN-Fen
PI distribution
PI distribution
PI distribution

## Slide 2
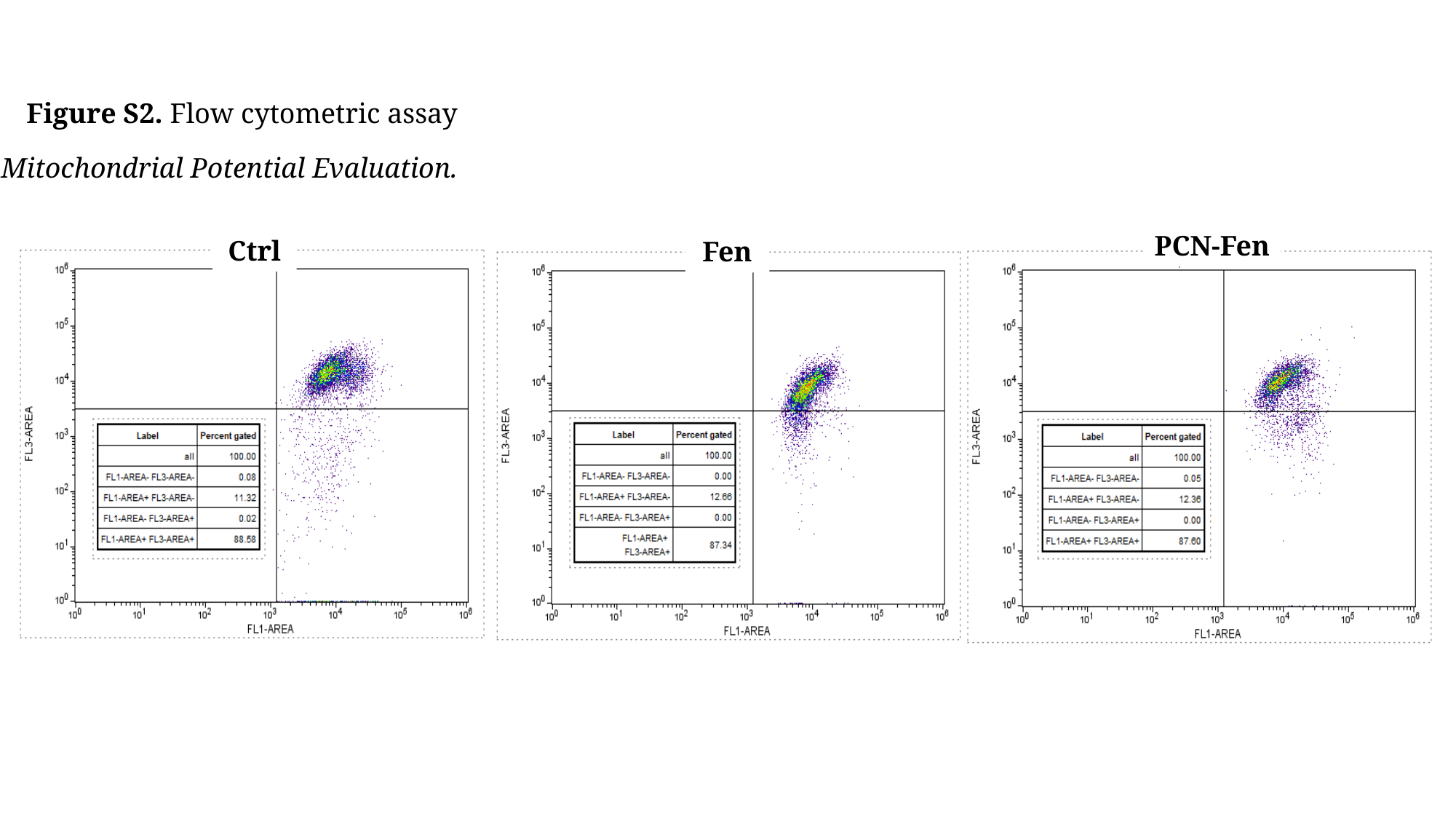

Mitochondrial Potential Evaluation.
PCN-Fen
Ctrl
Fen
Figure S2. Flow cytometric assay

## Slide 3
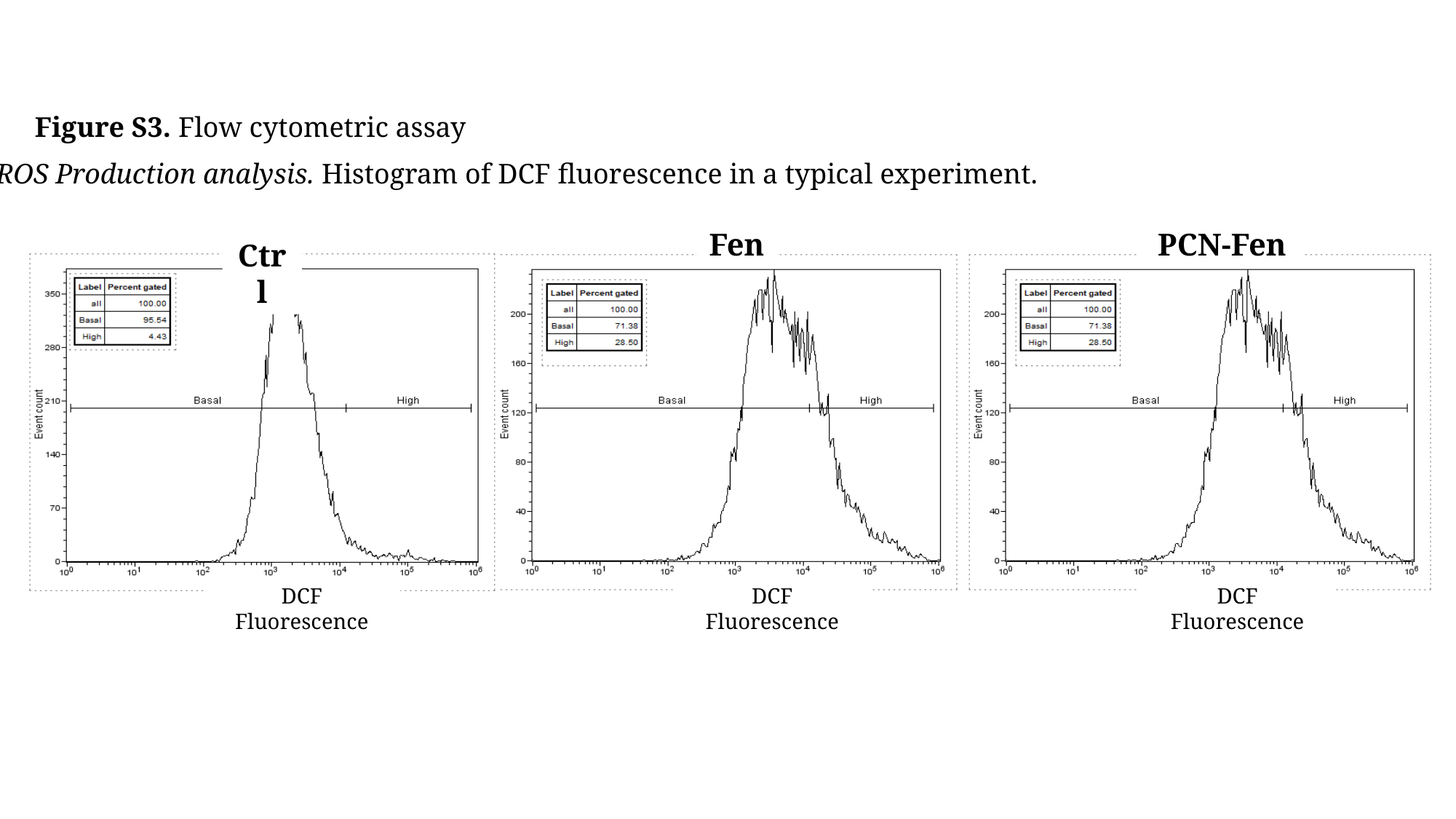

ROS Production analysis. Histogram of DCF fluorescence in a typical experiment.
Fen
PCN-Fen
Ctrl
DCF Fluorescence
DCF Fluorescence
DCF Fluorescence
Figure S3. Flow cytometric assay

## Slide 4
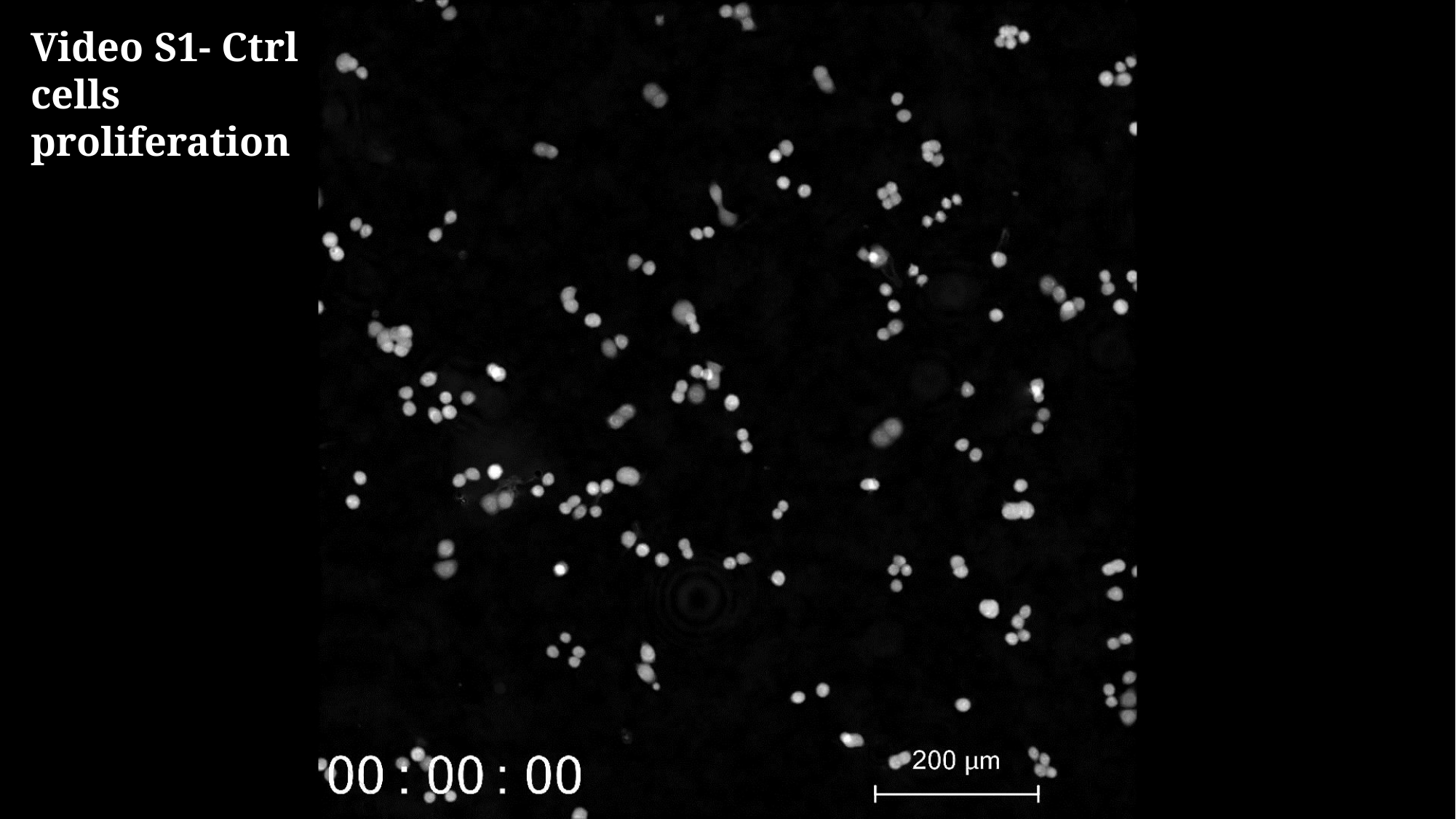

Video S1- Ctrl cells proliferation

## Slide 5
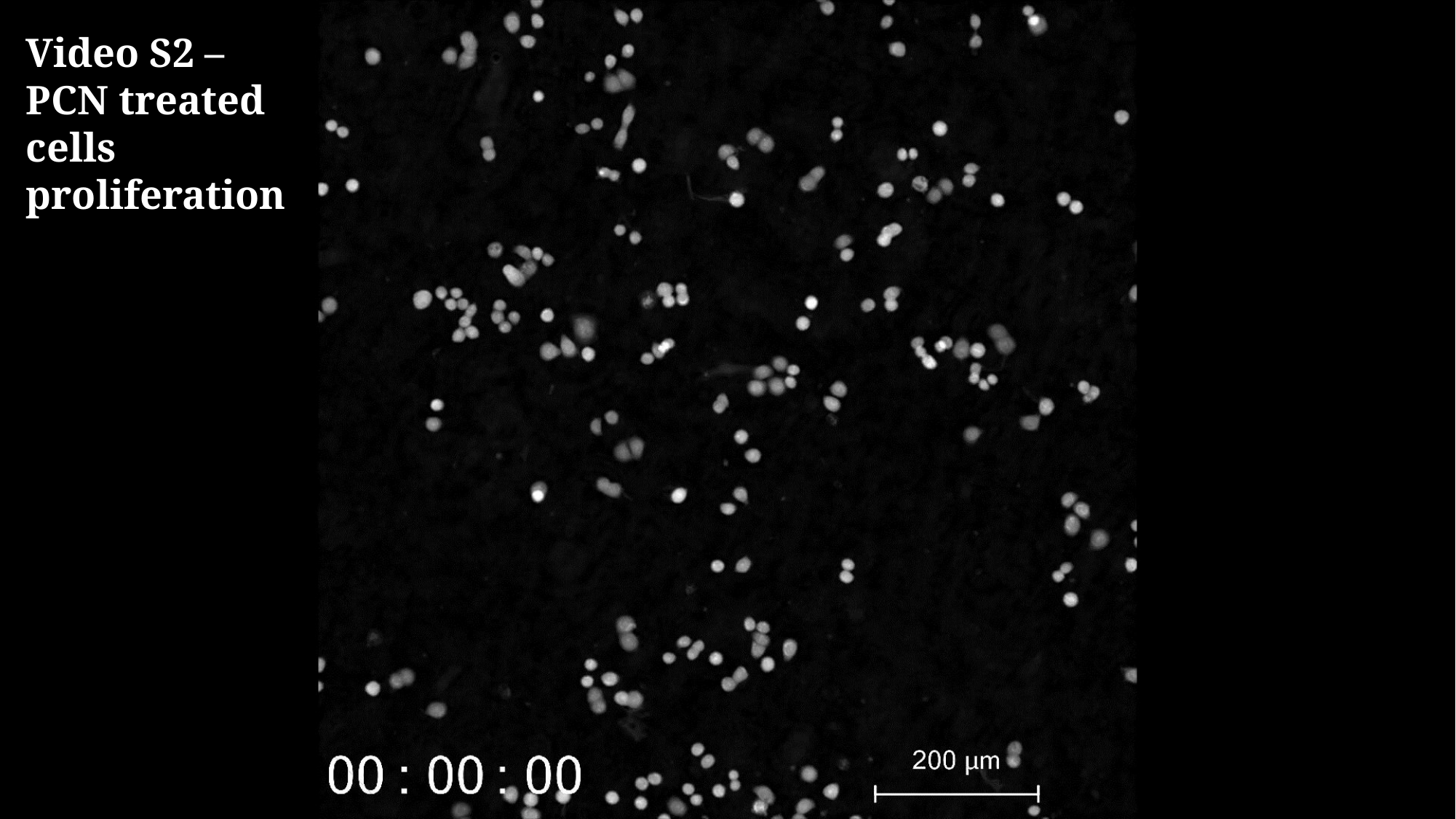

Video S2 – PCN treated cells proliferation

## Slide 6
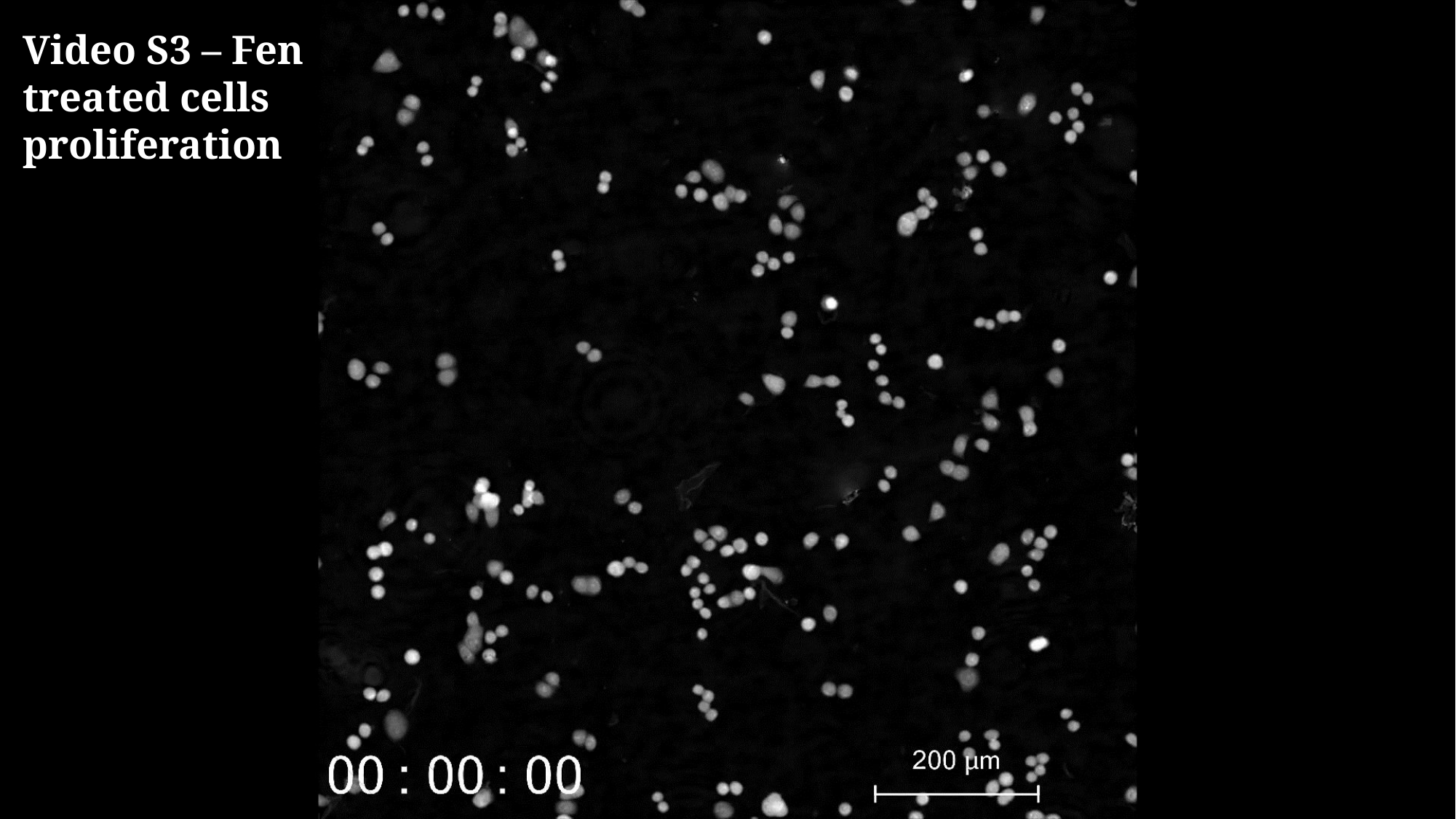

Video S3 – Fen treated cells proliferation

## Slide 7
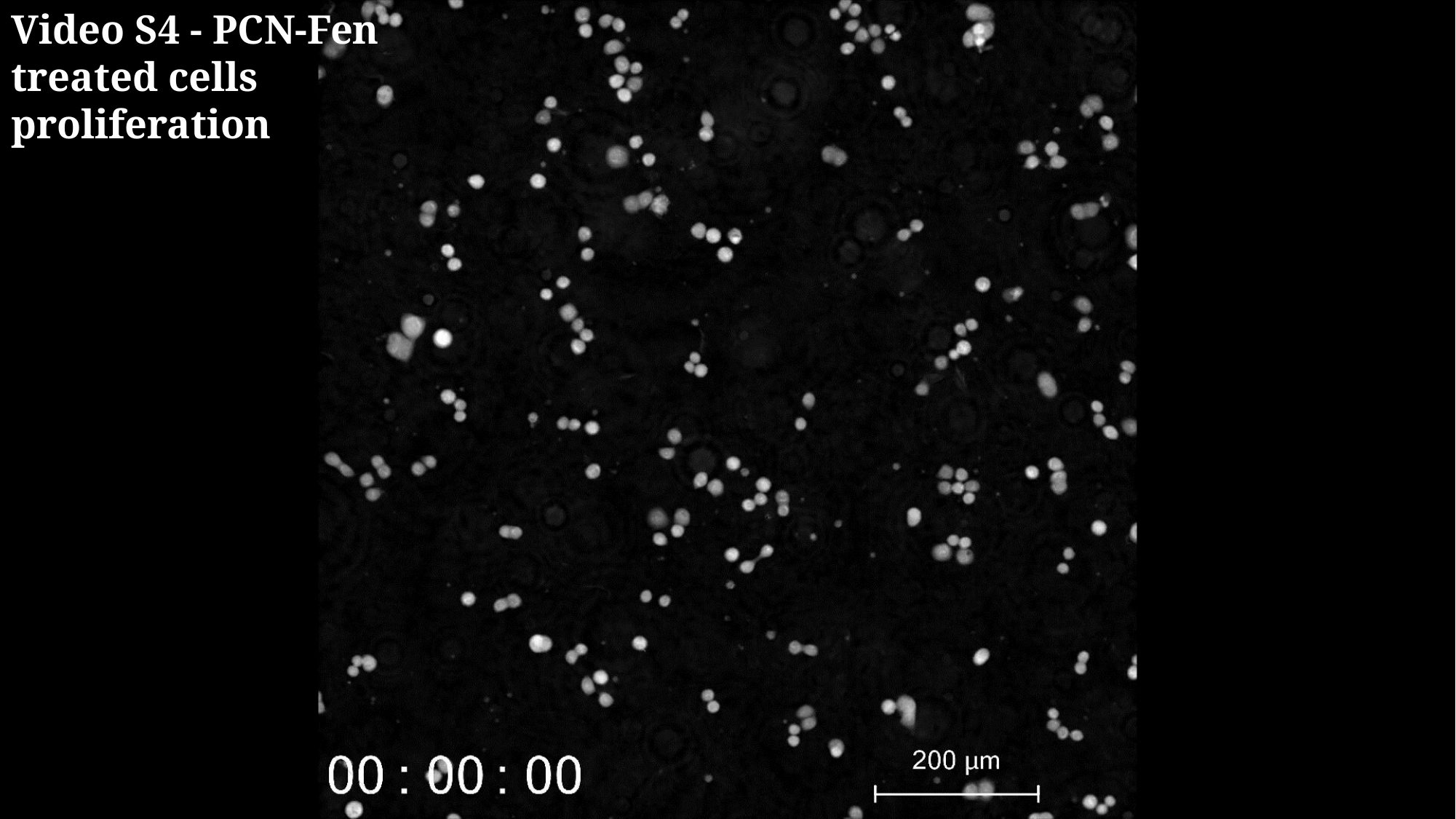

Video S4 - PCN-Fen
treated cells proliferation
